# Stereotactic Ablative Radiotherapy of Ventricular Tachycardia Using Tracking: Optimized Target Definition Workflow

**DOI:** 10.3389/fcvm.2022.870127

**Published:** 2022-05-02

**Authors:** Pavel Dvorak, Lukas Knybel, Denis Dudas, Pavla Benyskova, Jakub Cvek

**Affiliations:** ^1^Department of Oncology, University Hospital Ostrava, Ostrava, Czechia; ^2^Department of Radiation Protection, General University Hospital Prague, Praha, Czechia; ^3^Department of Oncology, University Hospital Motol, Praha, Czechia; ^4^Faculty of Medicine, University Hospital Ostrava, Ostrava, Czechia

**Keywords:** stereotactic, radiotherapy, target definition, motion management, tracking, deformation, Cyberknife

## Abstract

**Background and Purpose:**

Stereotactic arrhythmia radioablation (STAR) has been suggested as a promising therapeutic alternative in cases of failed catheter ablation for recurrent ventricular tachycardias in patients with structural heart disease. Cyberknife^®^ robotic radiosurgery system utilizing target tracking technology is one of the available STAR treatment platforms. Tracking using implantable cardioverter-defibrillator lead tip as target surrogate marker is affected by the deformation of marker–target geometry. A simple method to account for the deformation in the target definition process is proposed.

**Methods:**

Radiotherapy planning CT series include scans at expiration and inspiration breath hold, and three free-breathing scans. All secondary series are triple registered to the primary CT: 6D/spine + 3D translation/marker + 3D translation/target surrogate—a heterogeneous structure around the left main coronary artery. The 3D translation difference between the last two registrations reflects the deformation between the marker and the target (surrogate) for the respective respiratory phase. Maximum translation differences in each direction form an anisotropic geometry deformation margin (GDM) to expand the initial single-phase clinical target volume (CTV) to create an internal target volume (ITV) in the dynamic coordinates of the marker. Alternative GDM-based target volumes were created for seven recent STAR patients and compared to the original treated planning target volumes (PTVs) as well as to analogical volumes created using deformable image registration (DIR) by MIM^®^ and Velocity^®^ software. Intra- and inter-observer variabilities of the triple registration process were tested as components of the final ITV to PTV margin.

**Results:**

A margin of 2 mm has been found to cover the image registration observer variability. GDM-based target volumes are larger and shifted toward the inspiration phase relative to the original clinical volumes based on a 3-mm isotropic margin without deformation consideration. GDM-based targets are similar (mean DICE similarity coefficient range 0.80–0.87) to their equivalents based on the DIR of the primary target volume delineated by dedicated software.

**Conclusion:**

The proposed GDM method is a simple way to account for marker–target deformation-related uncertainty for tracking with Cyberknife^®^ and better control of the risk of target underdose. The principle applies to general radiotherapy as well.

## Introduction

Stereotactic arrhythmia radioablation (STAR) has been suggested as a promising therapeutic alternative in cases of failed catheter ablation for recurrent ventricular tachycardias (VTs) in patients with structural heart disease (1–4). Various radiotherapy treatment modalities are available for STAR. Each modality is associated with a technology and workflow-specific target definition process (2, 5, 6) with consequences to treatment efficacy and toxicity. STAR-specific combinations of cardiac and respiratory motions present challenging conditions for safe and accurate treatment.

The general principles of radiotherapy apply also to STAR. This includes acquiring a 3D planning CT scan of a patient comfortably placed and/or immobilized in the treatment position and allowing free access of radiation beams to deliver the treatment dose. The planning CT scan is then used as a 3D patient's model to define target volume(s) and critical organs in the vicinity of the target by computerized delineation and, after applying dose prescription and constraints, to optimize and calculate the final deliverable dose distribution. This procedure is known as radiation treatment planning and is carried out using dedicated computers and software known as *Treatment Planning Systems* (TPS). As the 3D patient's model in principle represents only a snapshot in time, i.e., excluding information about variations of anatomy during treatment delivery, various *motion management* approaches apply. These approaches differ in complexity, accuracy, technological demand, and, mostly, in the definitive treated volume to cover the whole range of assumed target motion. Simplified target volume concepts are as follows: the *clinical target volume* (CTV) indicates the 3D volume to treat, the *internal target volume* (ITV) is the CTV expanded by a known or estimated range of internal motion due to physiological processes such as respiration, and *planning target volume* (PTV) is the final volume to cover all remaining geometrical uncertainties to avoid missing (underdosing) any part of the CTV.

Cyberknife^®^ (Accuray Inc, Sunyvale, CA, USA) is a stereotactic radiotherapy dedicated treatment platform based on a 6MV X-ray medical linear accelerator mounted on an industrial robotic arm, a 4D or 6D robotic treatment couch as patient support during treatment, and two X-ray imaging systems for target localization before and during treatment. The prescribed treatment dose is delivered to the patient *via* dozens (typically 50–150) of radiation beams directed in a patient in a generally non-coplanar non-isocentric geometry. Collimator systems define the aperture of a group of radiation beams representing a key technological feature to conform the dose distribution to the target while minimizing the dose to the surrounding healthy tissue particularly the more sensitive structures known as organs at risk (OARs). One of the key features of the system is the ability to track. The tracking mode relevant to STAR is Synchrony using “fiducials.” Based on a target surrogate (in the case of STAR a selected *implantable cardioverter-defibrillator* (ICD) lead tip ideally close to the target), a correlation respiratory motion model is created before the treatment based on the ICD lead's 3D locations extracted from a series of X-ray image pairs and corresponding respiratory phase signal from LED markers placed on the patient's chest. The created model is used during dose delivery to control the radiation source position and orientation to move in synchrony with the target (surrogate) while the beam is on. During treatment, the correlation model is updated with every subsequently acquired new pair of X-ray images. By making use of three or more non-colinear markers, the system has the capability of tracking in 6D (3D translational + 3D rotational axes). In the case of STAR applications with a single marker, tracking is limited to 3D translations. In principle, this technology requires minimum target volume expansion for covering respiratory motion-related target position variation during breathing. Therefore, in theory, it is relatively more effective in sparing normal tissue from dose than standard clinical applications. On the other hand, there is a principle inconsistency between radiation treatment planning and treatment delivery. A treatment plan is created on a static single selected phase (typically expiration breath hold) CT model while treatment itself is performed at free breathing. There are two associated uncertainties: (a) the dose calculation and resulting optimized dose distribution are based on a limited CT model neglecting potential changes due to different tissue distribution at complementary breathing phases, and (b) the potential variation of mutual geometry between the target surrogate (“visible” by the system) and the target itself. The first aspect, that is the variation of dose distribution, is generally considered insignificant especially for a large number of treatment beams, range of beam directions, long beam delivery times compared to the breathing period and the resulting compensations of minor under- and over-doses. However, the second aspect represents a potential failure of the critical indirect tracking condition, i.e., the assumed fixed geometry bound between the tracked target surrogate and the target itself. In this work, we focus on investigating the magnitude of target surrogate (marker)–target geometry deformation and proposing a simple method using the Cyberknife^®^ system default equipment to compensate for associated uncertainty in target coverage through individual asymmetric margins to create ITV (in dynamic coordinates of tracked ICD lead tip) and final PTV for dose distribution optimization. This is the key aspect of the proposed target definition workflow improvement.

## Materials and Methods

### Original Workflow

Our treatment planning and delivery procedure have been described in detail previously (4, 7–9). After initial development, the original target definition workflow was established as follows:

- primary planning CT series (CT model for dose optimization and calculation): expiration breath hold CT (*CTebh*), full range for treatment planning.
- secondary CT image series acquired during one CT exam (CT study).
  ○ IV contrast expiration (shorter range) CT for electro-anatomical mapping and CTV definition (*CTebh-ivc*).
  ○ (natural) inspiration (shorter range) CT to sample cardiac and respiratory motion (*CTibh*).

Target definition is based on the 3D maps from the electroanatomic mapping system (CARTO, Biosense-Webster, Israel) (8, 10–12). Either endocardial or epicardial mapping points are acquired during the ablation process. Points are acquired in systole and expiration breathing phase controlled by respiratory phase monitor, so that the resulting 3D surface for registration with the reference radiotherapy target definition *CTebh-ivc* involves minimum uncertainty in position produced by breathing and cardiac motion. Sometimes, specific points that indicate scar region are identified by a cardiologist during the ablation process. In such cases, 3D image registration is driven also by points (“markup to points”). The “Rigid body” registration process is made in 3D Slicer software (13) and involves heart segmentation on the reference *CTebh-ivc*. Transferred points-voxels are “burned” in the reference *CTebh-ivc* image by associating with a high intensity (voxels with high Hounsfield units). This modified secondary CT image series is imported back in the TPS and registered with the primary planning *CTebh* series to form the base for the CTV. The radiation oncologist working concurrently with the cardiologist may change or correct the volume based on a detailed assessment of the anatomy and any complementary information. An additional isotropic 3-mm CTV-PTV margin is added to account for mainly LED signal—marker position correlation uncertainty, marker position—target position fixed bound uncertainty, and residual motion and technological uncertainty. The secondary image series are used to assess OAR relative to the target position at extreme respiratory phases and to provide contrast for indicating ventricle volume for CARTO-based target definition.

The dose distribution is optimized and calculated using Multiplan^®^ TPS (Accuray, Inc., Sunnyvale, CA, USA). The required coverage is for ≥ 95% of the PTV to receive 25 Gy, with the prescribed dose as close as possible to the 80% (of the global dose maximum) isodose line. The end of the right ventricular septal ICD lead is used as the surrogate marker and is continuously tracked with the target locating system of the Cyberknife^®^. Live images are acquired every 60 s (minimum) during treatment, and the correlation model is continuously updated. As the Synchrony system does not allow compensating rotations during treatment when only a single marker is being used, rotations of the body are eliminated during an initial patient setup based on an additional dummy spine-align treatment plan where spine in the target region is aligned to within 1°/1 mm from the CT model using the Xsight Spine tracking mode. This, however, does not compensate for target internal rotations which are an additional possible source of uncertainty and must be accounted for by the PTV margin. After spine alignment, the patient is moved to the treatment center with a robotic couch. The first pair of live images are acquired in the same breathing phase as in the digitally reconstructed radiographs (DRRs) generated from the planning CT scan. Patient alignment at the treatment center is verified using visible structures in the image, e.g., ICD lead, chest wall, diaphragm, stainless steel wires in the patient's chest, and spine structures.

### Improved Target Definition Workflow Description

The proposed target definition workflow improvement consists in addressing deformation of mutual geometry between the marker, i.e., ICD lead tip, and target volume during dose delivery at free breathing. One of the key attributes of the suggested method is its simplicity. The new target definition workflow is described as follows:

- primary planning CT series (CT model for dose optimization and calculation): expiration breath hold CT (*CTebh*), full range for treatment planning.
- secondary CT image series acquired during one CT exam.
  ○ IV contrast at expiration (shorter range) CT for electro-anatomical mapping and CTV definition (*CTebh-ivc*).
  ○ (natural) inspiration (shorter range) CT to sample cardiac and respiratory motion (*CTibh*).
  ○ 3x native free-breathing CT (shorter range) to sample cardiac and respiratory motion (*CTfb1-3*).

**Figure 1:**
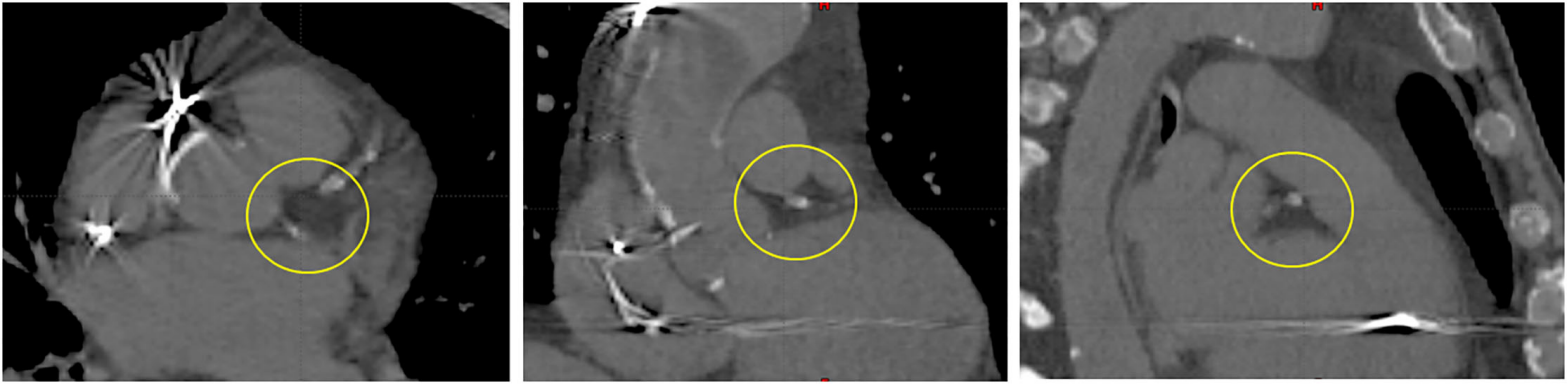
LMCA region (circled)—a visible heterogeneity used as the primary target surrogate inside a heart (RefSTRUCT). From left to right: transverse, coronal, and sagittal view.

The purpose and parameters of the primary planning *CTebh* and also the first secondary *CTebh-ivc* remain the same. Notwithstanding the fact that both *CTebh* and *CTebh-ivc* are acquired at the same respiratory phase and the use of respiratory monitor to control reproducibility, there may be some misregistrations between the two image series. As previously mentioned, *CTebh-ivc* is used for primary CTV definition using CARTO mapping. Transferring this volume to the primary planning CT model (*CTebh*) without introducing uncertainty requires a registration check of the secondary image based on the cardiac anatomy particularly in and around the target region. The remaining four secondary CT series (*CTibh, CTfb1-3*) are used for both cardiac and respiratory motion assessment with respect to the variability of relevant anatomy relative to the marker tracked by the system during treatment delivery at free breathing. This means that after importing all image series in the TPS all but the *CTebh-ivc* are first 6D registered to the spine as motion intact anatomy used for the patient's initial pretreatment setup mainly to eliminate rotations not accounted for during single marker tracking. Subsequently, these are registered 2nd time to the marker. Since the marker is tracked by the system, visible variations of anatomy on the secondary images now represent parasite motion (rotations and deformations) which would need to be accounted for by appropriate target expansion—ITV (in dynamic coordinates of the tracked marker). Since the anatomy landmarks in the target region are difficult to distinguish on a CT image, it would be possible to expand the original CTV simply by contouring. We developed a simple procedure using a clear heterogeneous structure inside the heart as a target surrogate in terms of marker–target geometry. This reference structure *RefSTRUCT*, shown in Figure 1, is the area around the *left main coronary artery* (LMCA).

To quantify motion for the needed compensation, we record 3D translation coordinates stored by the TPS in the image registration transform matrix after initial 6D/spine + 3D/marker registrations. In the next step, we perform the 3rd registration-−3D translation to the *RefSTRUCT* for each secondary image used for motion assessment (3D/*RefSTRUCT*). Then, we record the changed 3D translation coordinates from the transform matrix. The difference in each direction represents the associated change of marker–target (surrogate) geometry to be used to expand the original CTV for eliminating the known risk of target underdose during free-breathing treatment delivery. For the final ITV definition, the maximum detected difference from all four motion assessment CT image series in six major anatomical directions (anterior, posterior, right, left, superior, and inferior) is used to expand the original CTV by this generally anisotropic margin—*geometry deformation margin* (GDM).

For final PTV, an additional isotropic margin of 2 mm is added to compensate mainly LED signal—marker position correlation uncertainty, intra- and inter-observer variabilities as the major sources of uncertainty of the GDM method, and residual motion and technological uncertainty.

Treatment planning data of a total of seven recent patients (2019–2021, 5 men, 2 women, various ages, and conditions) treated with STAR at our institution were analyzed retrospectively to create an alternative target volume (ITV_GDM_ and PTV_GDM_) following the proposed improved target definition workflow. For all patients, the clinically applied, i.e., treated, PTV was created using the original workflow described in the previous section. However, as a part of the planning CT acquisition, all patients underwent additional CT imaging (2 or 3 shorter range free-breathing CT series) to sample cardiac and respiratory motion making the data coverage equivalent to the improved target definition workflow. Retrospectively, created ITV_GDM_ and PTV_GDM_ volumes were imported in Eclipse^®^ TPS (version 13.6, Varian Medical Systems Inc., Palo Alto, CA, USA) for analysis with respect to the objectives of this study.

### Improved Workflow Verification

Although the principle of the suggested target definition workflow, based on the semi-automated (6D/spine) and individual manual rigid registration (3D/marker + 3D/*RefSTRUCT*) of secondary image series often with significant metal image and motion artifacts is clear, the outcomes are dependent on intra- and inter-observer variabilities. Within the described workflow, the estimated intra- and inter-observer variabilities of the subjected manual image registration should form the basis for the residual motion uncertainty component of the PTV margin.

In principle, the investigated motion aspect is the deformation of the mutual marker–target geometry during free breathing. With the primary CTV based on CARTO mapping on CT at expiration, it is logical to consider the primary CTV expansion through an application of deformable image registration (DIR) of underlying CT images acquired at complementary respiratory phases (*CTibh, CTfb1-3*) to cover fully the sampled range of motion at free breathing. The merging of the primary CTV structure deformed on the background of all four complementary CT images forms a technique equivalent to an ITV constructed using GDM as described above. A number of two dedicated state-of-the-art software products, MIM (MIM^®^ software Inc., Beachwood, OH, USA) and Velocity^®^ (version 4, Varian Medical Systems Inc., Palo Alto, CA, USA), were used to compare ITVs obtained using the proposed simple method based on GDM (ITV_GDM_) with ITV equivalents ITV_VELO_ and ITV_MIM_ obtained using DIR.

#### Intra- and Inter-Observer Variabilities

All available secondary CT series acquired for motion management purposes (i.e., *CTibh, CTfb1-2* or *CTfb1-3*) underwent a full sequence of manual image registrations (6D/spine + 3D/marker + 3D/*RefSTRUCT*). Relevant parameter values from the image registration transform matrix for five recent patients—a subgroup of seven used for ITV_GDM_ testing—were recorded. Each image registration was repeated 3 times (not consecutive runs) by each of the two observers. A total of two metrics were chosen for the comparisons:

- DICE similarity coefficient representing the similarity of two 3D volumes (14), in this case, _*i*−*j*_*ITV*_*GDM*_ where *i* = 1,2 (observers performing registrations), j = 1,2,3 (number of tests performed).
- Hausdorff average distance (H-AVE) indicating mean distance between each point of one compared structure to the closest point in the other structure (15). The reason for using also H-AVE is mainly because it is closely related to a size of margin in mm to cover observed uncertainty—major expected outcome from intra- and inter-observer variation tests.

For intra-observer variability investigation, each patient, and each of two observers, altogether, three mutual comparisons were made, i.e., _1−j_ITV_GDM_ and _2−j_ITV_GDM_, respectively (j = 1,2,3), giving 6 measurements per patient in total.

For inter-observer variability investigation, each patient, each secondary image series, and each of 3 tests by observer 1, comparisons with results of equivalent tests by observer 2 were made, i.e., _i−j_ITV_GDM_ and _i−j_ITV_GDM_, respectively (*i* = 1,2, j = 1,2,3), giving 9 measurements per patient in total.

#### DIR Using MIM^®^

All relevant secondary CT series (*CTibh, CTfb1-2* or *CTfb1-3*) registered (6D/spine + 3D/marker) previously in Multiplan^®^ TPS together with original primary planning CT series (*CTebh*) and associated original structure set (RS) selection including original CTV were imported in MIM^®^ software for each patient. Using standard *AdaptiveRecontour–Deform* workflow, we DIR-registered the planning *CTebh* + RS to each of 3 or 4 secondary CT series. During the workflow run, the initial rigid registration was reset to maintain the original registration on the marker. The registration products, i.e., deformed planning *CTebh* and deformed RS were saved. In the following step, the deformed *CTebh* was opened together with the original DIR target image (*CTibh, CTfb1-2*, or *CTfb1-3*), and using the image fusion mode and tools, the quality of DIR was checked focussing on the area of the marker and *RefSTRUCT*. Finally, depending on a patient, three or four deformed primary CTV structures together with deformed *CTebh* images were exported from MIM ^®^ and imported in Eclipse^®^ TPS as components of ITV_MIM_ volume and for further analysis.

#### DIR Using Velocity^®^

Deformable registrations carried out in Velocity^®^ used the same inputs as MIM^®^. The images were then initially manually registered according to the marker using a rigid transformation. Following this, a DIR was performed inside a region of interest which was set with a 1-cm margin around the heart. The employed DIR uses a modified B-spline deformable algorithm with mutual information metric for the evaluation of similarity between registered images (16). Other available algorithms were either not suitable for given CT series or produced significant image artifacts. As with MIM^®^, DIR products and, depending on the patient, three or four deformed primary CTV structures together with the deformed *CTebh* images were exported from Velocity^®^ and imported in Eclipse^®^ as the components of ITV_VELO_ volume and for further analysis.

For both MIM^®^ and Velocity^®^ registration products, sometimes, the DIR process moved the marker within the CT series, so the marker centered registration had to be slightly adjusted.

### Mutual Comparisons

**Figure 2:**
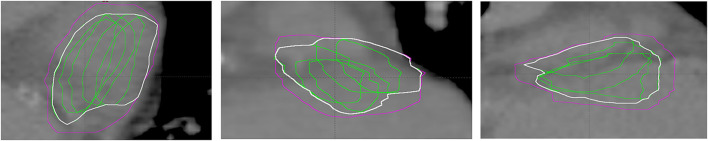
Example of ITV_GDM_ based on maximum detected translation (magenta) vs. ITV_GDM−SUM_ (white) based on merging motion sampling subvolumes (green). From left to right: transverse, coronal, and sagittal view.

For each patient, the final sets of structures for data analysis in Eclipse^®^ contain the original CTV, original PTV, volumes created by methods described above, ITV_GDM_, ITV_VELO_, ITV_MIM_, and the derived PTV volumes obtained by the isotropic expansion of the respective ITV volume by 2 mm (PTV_GDM_, PTV_VELO_, and PTV_MIM_, respectively). In addition, an extra ITV_GDM−SUM_ volume was created as an alternative to the GDM method (based on the maximum translational difference of all secondary images in each of the six major cardinal directions) by merging all secondary image registration-related subvolumes, i.e., an analogous method as applied for the ITV_MIM_ and ITV_VELO_ (refer to Figure 2 for an example of the difference.) For a subgroup of five patients, an additional reference structure, *RefSTRUCT*+, was used as a target surrogate in the last 3D translational registration to determine GDM components. The reason was that this anatomy (less clear but still well identifiable) was significantly closer to the target. Therefore, two versions of ITV and resulting PTVs were included for this subgroup. Definitive sets of structures for mutual comparisons are PTV, _1_PTV_GDM_, _1_PTV_GDM−SUM_, PTV_MIM_, PTV_VELO_ (for all patients), and _2_PTV_GDM_ and _2_PTV_GDM−SUM_ (for the subgroup of 5 patients).

With regard to mutual comparisons of created structures, four objectives were defined:

1. What is the difference between the original treated PTV volume and the alternative PTV_GDM_? if volumes are comparable, there is a question whether the more laborious workflow is justified. If a GDM volume is larger, there is the question of the relative increase in potential toxicity of treatment (assuming the same target coverage).
2. is PTV_GDM_ (or rather PTV_GDM−SUM_) similar to volumes obtained using DIR (PTV_MIM_ and/or PTV_VELO_)? if it is then this can be interpreted as mutual validation of the GDM principle, i.e., a simple GDM method is validated by a clinical standard DIR software product(s).
3. What is the difference between PTV_GDM_ based on *RefSTRUCT* and *RefSTRUCT*+ target surrogates for situations where distance between marker and target is larger? if the difference is small, then marker–target geometry deformation is more likely to be described by the default universal *RefSTRUCT* even for larger marker–target distances.
4. What is the difference between PTV_GDM_ and PTV_GDM−SUM_ to compare approaches based on the maximum observed translation (GDM) and based on the merge of 3D translational difference subvolumes? If the difference exists, then the GDM approach is preferred because it has the principal advantage of not missing parts of target due to (continuous respiratory) motion undersampling.

A number of two metrics were selected for mutual comparisons of volumes:

- absolute volumes in cm^3^.
- DICE similarity coefficient for each subjected pair of volumes compared.

The statistics were calculated using the software STATISTICA 13 (TIBCO Software Inc., Palo Alto, CA, USA). All quantitative data were expressed as mean±SD. Paired *t*-tests were used to distinguish between two paired sets of measurements. All tests were performed at the 5% level of significance.

## Results

### Intra- and Inter-Observer Variabilities

Intra-observer variability results for reproducibility of triple image registration (GDM method) in terms of both DICE coefficient and H-AVE are presented in Table 1. Inter-observer analogy is summarized in Table 2.

**Table 1:** Intra-observer variability results (DICE and H-AVE) of mutual comparisons of ITV_GDM_ volumes after triple image registration made by 2 observers (3 tests for each of 5 patients).

**Vol. 1**	**vs. Vol. 2**	**DICE**					**H-AVE [mm]**				
		**1**	**2**	**3**	**4**	**5**	**1**	**2**	**3**	**4**	**5**
_1−j_ITV_GDM, j = 1, 2, 3_	_1−j_ITV_GDM, j = 1, 2, 3_	0.83	0.84	0.91	0.92	0.86	1.6	2.1	0.7	0.8	1.2
		0.84	0.87	0.91	0.87	0.88	1.6	1.6	0.8	1.5	1.1
		0.88	0.92	0.90	0.87	0.91	1.1	1.0	0.8	1.4	0.8
_2−j_ITV_GDM, j = 1, 2, 3_	_2−j_ITV_GDM, j = 1, 2, 3_	0.91	0.97	0.90	0.76	0.95	0.9	0.4	0.9	2.3	0.5
		0.94	0.91	0.98	0.69	0.92	0.7	1.3	0.2	3.1	0.7
		0.92	0.90	0.89	0.87	0.97	0.8	1.4	1.0	1.6	0.2
	mean =	0.89					1.1				

**Table 2:** Inter-observer variability results (DICE and H-AVE) of mutual comparisons of ITV_GDM_ volumes after triple image registration made by 2 observers (3 tests for each of 5 patients).

**Vol. 1**	**vs. Vol. 2**	**DICE**					**H-AVE [mm]**				
		**1**	**2**	**3**	**4**	**5**	**1**	**2**	**3**	**4**	**5**
_1−1_ITV_GDM_	_2−j_ITV_GDM, j = 1, 2, 3_	0.86	0.87	0.92	0.82	0.88	1.4	1.4	0.6	1.7	1.1
		0.87	0.94	0.87	0.83	0.95	1.2	0.8	1.1	1.7	0.4
		0.85	0.89	0.91	0.88	0.90	1.5	1.4	0.8	1.3	0.9
_1−2_ITV_GDM_	_2−j_ITV_GDM, j = 1, 2, 3_	0.83	0.88	0.85	0.84	0.90	1.7	1.5	1.3	1.6	0.9
		0.86	0.90	0.90	0.78	0.88	1.5	1.3	0.8	2.1	1.1
		0.84	0.93	0.95	0.75	0.91	1.6	0.9	0.3	2.3	0.8
_1−3_ITV_GDM_	_2−j_ITV_GDM, j = 1, 2, 3_	0.87	0.86	0.98	0.80	0.98	1.3	1.9	0.2	1.9	0.2
		0.82	0.98	0.92	0.77	0.90	1.7	0.2	0.7	2.2	1.0
		0.89	0.97	0.91	0.91	0.93	1.1	0.4	0.8	2.1	0.7
	mean =	0.88					1.2				

**Figure 3:**
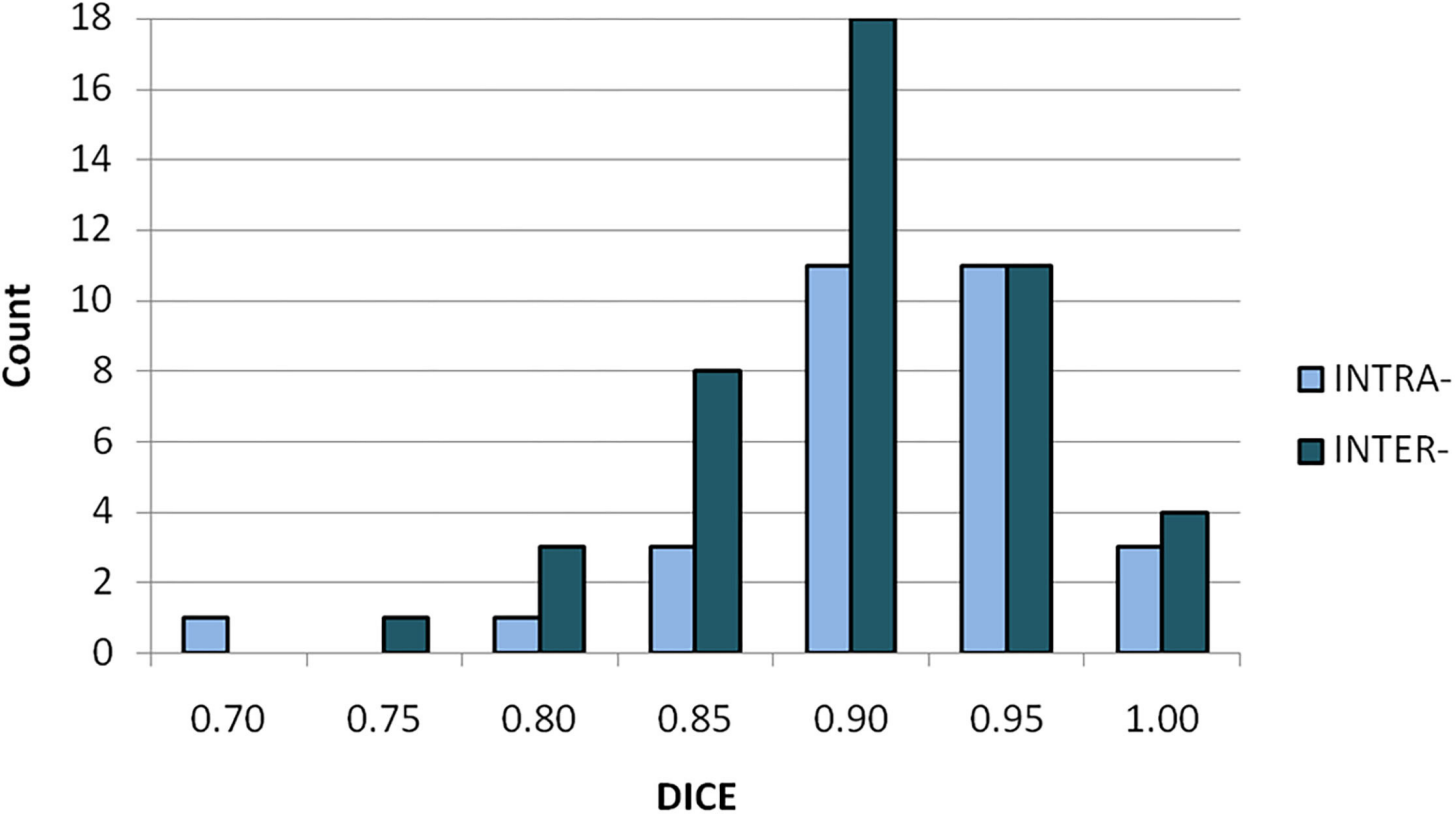
Differential histogram of DICE coefficients for intra- (light/blue) and inter-observer (dark/aquamarine) variabilities.

The differential histogram of DICE coefficients for both intra- and inter-observer variabilities is shown in Figure 3. The two histograms present variation of volume-comparison metrics with identical input, i.e., with an identical outcome expected. All tests showed DICE over 0.75; 95% (71/75) of tests showed DICE value over 0.8.

**Figure 4:**
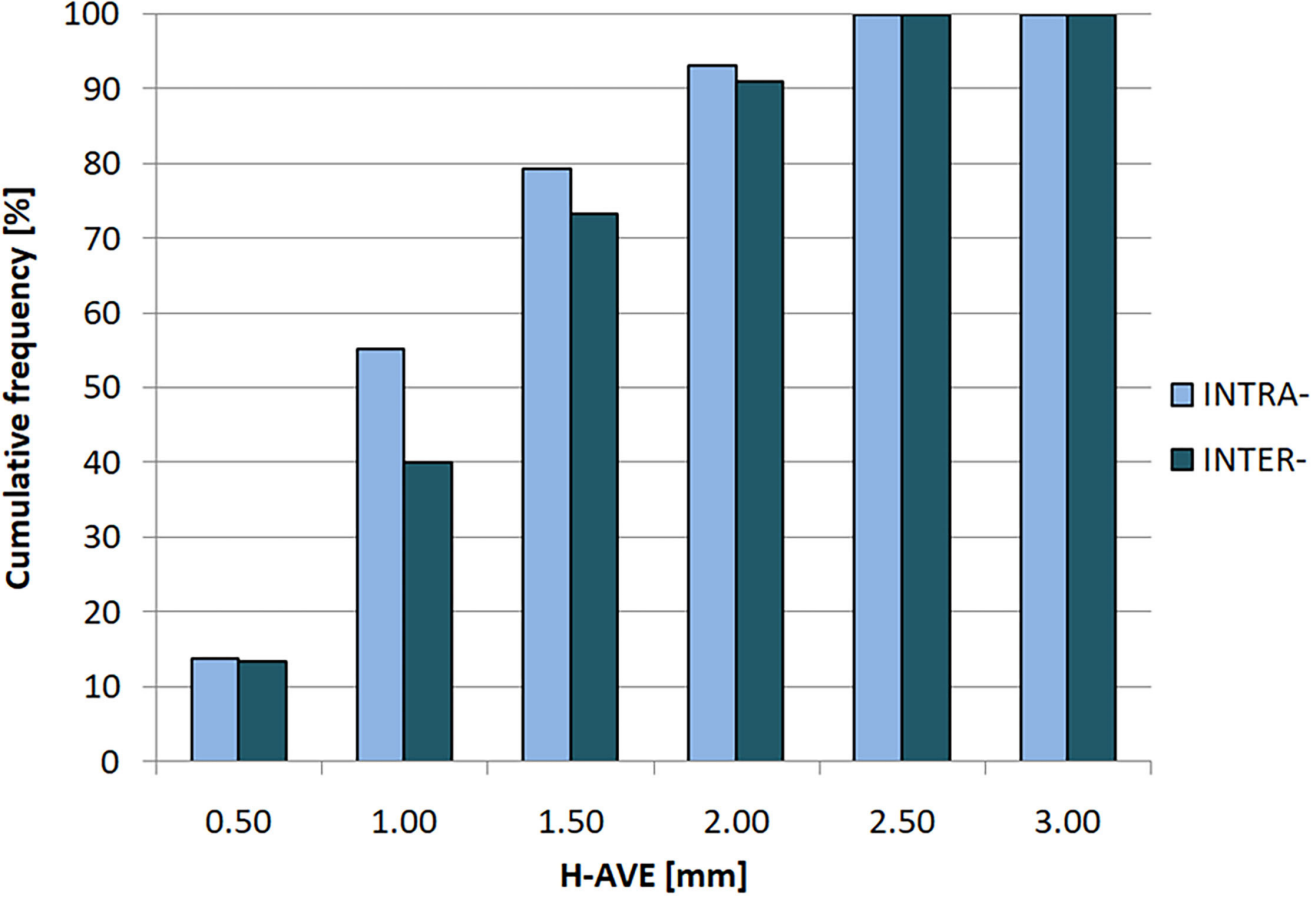
Cumulative histogram of H-AVE (Hausdorff average distance) for intra- (light/blue) and inter-observer (dark/aquamarine) variabilities.

Integral histogram of H-AVE values of both intra- and inter-observer tests is shown in Figure 4. In both categories of tests, 2 mm of margin covers observed volume variability in terms of image registration and selected volume-comparison metrics. This is an important estimate of image registration-related component of GDM method uncertainty that should be compensated for by additional margin to ITV_GDM_ to avoid target underdose.

### Mutual Comparisons

Table 3 shows absolute volumes of all relevant structures to demonstrate the differences across the given volume categories. The original (treated) volumes are always smaller than any alternative (GDM- and DIR-based) volumes. This demonstrates that deformation of marker–target geometry during free breathing is present and, based on the presented values, is not small. General increase in target volume is technically associated with the relative increase in treatment toxicity. To what extent this is relevant to STAR is the subject of the Discussion.

**Table 3:** Absolute volumes indicated by TPS for each constructed structure for 7 patients.

**Volume [cm^**3**^]**	**Patient No**						
	**1**	**2**	**3**	**4**	**5**	**6**	**7**
PTV	28.3	45.0	12.5	19.8	25.9	69.3	39.2
_1_PTV_GDM_	69.7	79.5	21.6	35.8	36.6	94.5	67.3
_2_PTV_GDM_	65.7	68.5	NA		42.7	87.4	73.4
_1_PTV_GDM−SUM_	50.4	63.5	17.5	30.1	34.0	86.3	53.0
_2_PTV_GDM−SUM_	45.8	58.6	NA		38.1	83.1	60.5
PTV_MIM_	44.1	102.7	17.5	24.7	41.2	97.9	62.6
PTV_VELO_	47.8	63.3	20.9	26.5	33.3	86.7	50.3

Volumes, constructed by merging subvolumes that are based on sampling location during motion, are generally smaller (GDM-SUM, MIM, and VELO) than volumes based on the original GDM method with maximum detected range of motion in six anatomical directions followed by appropriate volume expansion. This is in agreement with the expected undersampling of the volume location during motion (for demonstration refer to Figure 2). The same mechanism makes GDM-SUM volumes closer to DIR-based (MIM and VELO) volumes confirming it is this volume construction method that should be used for GDM vs DIR comparison purpose.

_2_PTV_GDM_ volumes do not appear larger than _1_PTV_GDM_ volumes generated by image registration to *RefSTRUCT* and *RefSTRUCT*+, respectively. This would be expected if there is a relative increase of deformation with increasing distance from the marker. This finding would support using primary *RefSTRUCT* as a possible universal target surrogate.

Results of the comparison of respective volumes in terms of DICE coefficient are presented in Table 4. There is a significant difference (*p* = 0.025) between DICE values for 1PTVGDM vs PTV (0.73 ± 0.08) and all other DICE values from Table 4 not related to comparisons with PTV (0.84 ± 0.05). The same applies to _2_PTV_GDM_ volumes based on *RefSTRUCT*+ with _2_PTV_GDM_ vs. PTV (0.74 ± 0.10). This supports the justification of the GDM method for the difference in final location, shape, and volume of target volume, assuming it better addresses motion-related uncertainty.

**Table 4:** Results of relevant volumes mutual comparison in terms of DICE coefficient for 7 patients.

**Vol. 1**	**vs Vol. 2**	**Patient No**							**Mean**	**StDev**
		**1**	**2**	**3**	**4**	**5**	**6**	**7**		
_1_PTV_GDM_	PTV	0.58	0.72	0.73	0.71	0.82	0.83	0.73	0.73	0.083
_2_PTV_GDM_	PTV	0.59	0.78	NA		0.75	0.87	0.70	0.74	0.103
_1_PTV_GDM_	_2_PTV_GDM_	0.86	0.87			0.92	0.94	0.87	0.89	0.036
_1_PTV_GDM_	PTV_MIM_	0.71	0.75	0.83	0.81	0.86	0.90	0.84	0.81	0.065
_1_PTV_GDM−SUM_	PTV_MIM_	0.77	0.71	0.89	0.85	0.84	0.90	0.85	0.83	0.068
_2_PTV_GDM_	PTV_MIM_	0.71	0.76	NA		0.85	0.88	0.77	0.79	0.069
_2_PTV_GDM−SUM_	PTV_MIM_	0.79	0.72			0.84	0.88	0.78	0.80	0.061
_1_PTV_GDM_	PTV_VELO_	0.80	0.84	0.84	0.84	0.90	0.92	0.80	0.85	0.046
_1_PTV_GDM−SUM_	PTV_VELO_	0.85	0.88	0.83	0.87	0.91	0.91	0.86	0.87	0.030
_2_PTV_GDM_	PTV_VELO_	0.77	0.86	NA		0.85	0.91	0.80	0.84	0.054
_2_PTV_GDM−SUM_	PTV_VELO_	0.80	0.86			0.87	0.90	0.86	0.86	0.036
PTV_MIM_	PTV_VELO_	0.85	0.73	0.90	0.92	0.84	0.87	0.81	0.85	0.063

*PTV = original treated volume, _1_PTV_GDM_ = based on GDM (maximum detected translation) and RefSTRUCT, _1_PTV_GDM−SUM_ = based on merging motion sampling subvolumes, PTV_MIM_ and PTV_VELO_ = based on merging motion sampling subvolumes from DIR by MIM/Velocity, and _2_PTV_GDM−SUM_, and _2_PTV_GDM_, analogically for RefSTRUCT+*.

Mean DICE values (range 0.80–0.87) for comparisons between PTV_GDM−SUM_ volumes and DIR-based, i.e., PTV_MIM_ and PTV_VELO_ volumes are relatively larger, comparable to mean DICE values for intra- (0.88) and inter-observer (0.89) variabilities where the “only” source of difference is reproducing work instruction for image registration and also to the guidelines recommended 0.8–0.9 to test DIR quality (17). The range of DICE values for individual patients and both _1_PTV_GDM−SUM_ and _2_PTV_GDM−SUM_ is 0.71–0.91 with 4 of 24 values below 0.8 for patients 1 and 2 and DIR based on MIM^®^. Minimum DICE value for DIR-based volumes using Velocity^®^ is 0.8. These results support the similarity between GDM-SUM and DIR-based volumes.

Mean DICE values for comparisons between _1_PTV_GDM_ and _2_PTV_GDM_ volumes are large (0.89, range 0.86–0.94) supporting the hypothesis that dominant deformation of marker–target surrogate geometry occurs mainly in the area between the marker and *RefSTRUCT*, making possible this main reference structure applicable even for targets more distant from the marker. This is also supported by the significant difference (*p* < 0.001) between DICE values for _1_PTV_GDM_ vs. _2_PTV_GDM_ (0.89 ± 0.04) and all other DICE values from Table 4 not related to comparisons with PTV (0.83 ± 0.05).

PTV_GDM−SUM_ volumes compared with PTV_MIM_ and PTV_VELO_ volumes show slightly larger (mean) DICE values compared to corresponding values for original PTV_GDM_ volumes; however, none of all relevant comparisons {(_1, 2_PTV_GDM_ vs. PTV_MIM, VELO_) vs. (_1, 2_PTV_GDM−SUM_ vs. PTV_MIM, VELO_)} showed any statistically significant difference, so the expected better agreement with GDM-SUM volumes is not statistically confirmed.

**Figure 5:**
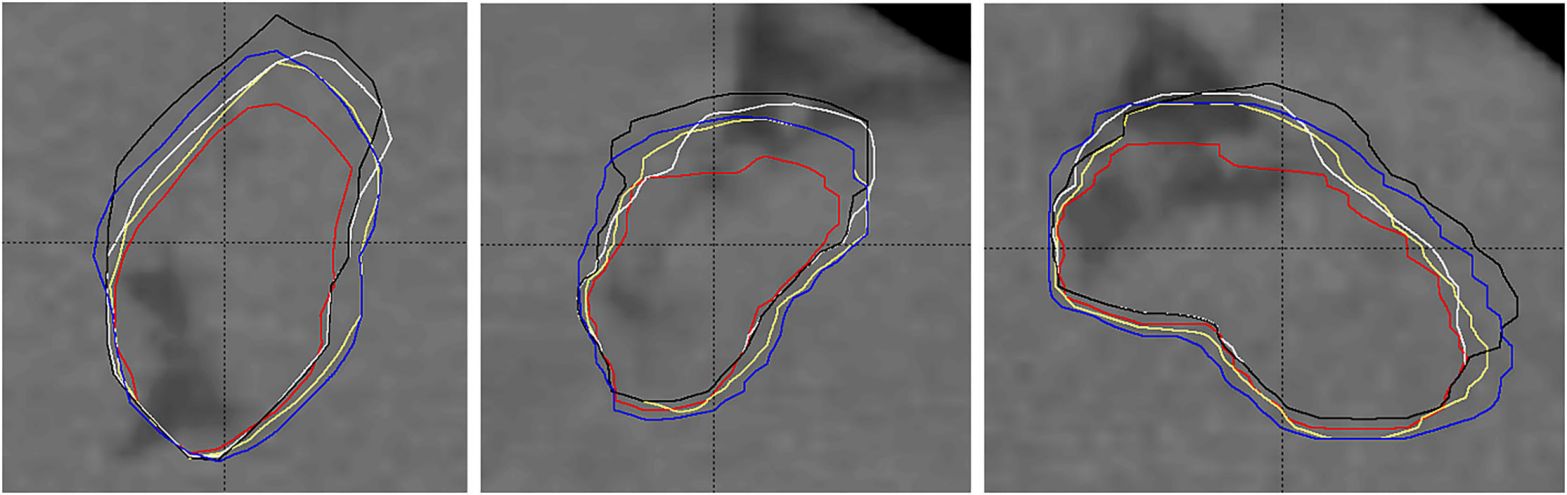
Example of planning target volumes: original treated PTV (red, smallest), PTV_GDM_ (blue), PTV_GDM−SUM_ (yellow), PTV_MIM_ (white), and PTV_VELO_ (black). 1) RefSTRUCT (LMCA) on background CT image used as target surrogate, 2) anisotropic extension of the original target toward inspiration, 3) lesser differences among GDM- and DIR-based volumes than their difference to the original PTV.

Figure 5 shows an example of the subjected target volumes for one patient. The expected shift toward inspiration phase CT data, larger volume, and better similarity among GDM and DIR-based targets (PTV_GDM_, PTV_GDM−SUM_, PTV_MIM_, and PTV_VELO_) compared to the original target (PTV) can be seen.

## Discussion

The main motivation for this study was to improve target definition workflow for STAR using Cyberknife^®^ target tracking technology by explicit consideration of deformation of the marker–target geometry present during treatment delivery as a result of cardiac and respiratory motion. To be applicable clinically, the workflow must not only be effective but also simple and robust to ensure efficiency while ideally relying on minimum extra resources. The proposed approach requires only essential equipment. In principle, the problem is solved using a “4D planning” approach (18). However, although 4D planning module on Accuray system has been available in the past, it is not offered as a feature in newer versions of TPS (Precision^®^, version 3.+) anymore, mainly because it was very rarely used in the clinical practice (19). The application of additional DIR capable software in the way applied to MIM^®^ and Velocity^®^ in this study would be a natural alternative to the proposed GDM method, which is associated with the need for additional resources and data transfers. Using DIR dedicated software to deform the initial CTV and merge would require uncertainty of DIR product assessment to replace the registration observer variability margin component applied in the PTV_GDM_. In general, DIR dedicated software may produce good results with time-saving but considering the complexity of the specific input data, i.e., CT series with motion and metal artifacts, this may not be a simple task as shown for example in Speight et al. (20) and Tong et al. (21). In addition, when using repeated free-breathing CT series to sample respiratory motion with consequent merging subvolumes, there is a risk of the total volume being smaller than adequate for risk of motion range undersampling as demonstrated, e.g., in Figure 2 or at GDM-SUM vs. GDM comparisons in this study. This represents an additional consideration for DIR-capable software alternative to the GDM method.

Considering these factors, the GDM method seems to be a simple and, based on this study, acceptable approximation of “4D planning” (in terms of geometry, not dosimetry) with the potentially important advantage of direct user control of the output.

### Study Limitations

The presented GDM method has several essential considerations. The first is a question of the representativeness of multiple CT image series to sample combined respiratory and cardiac motion. In particular, *CTibh* tends to be exaggerated when the patient is instructed or respiratory phase monitoring is suboptimal, so careful respiratory management of data acquisition and assessment is important. Although 4DCT is certainly an option, it is not currently available in our department and even with 4DCT, there is a question of representativeness and image quality including residual motion and metal artifacts. Nevertheless, the presented GDM is applicable to 4DCT as well.

The next principal question is the relevance of using LMCA area (and other contrast anatomy in heart) as yet another target surrogate. Nevertheless, based on the relatively large deformation observed (demonstrated also by the larger resulting PTV_GDM_ vs. PTV volumes) and the relatively small difference between volumes based on *RefSTRUCT* and *RefSTRUCT*+, the method can be justified as an adequate approximation bringing with it the benefit of considering deformation for target definition. For 5 of 7 patients, in addition to the LMCA (*RefSTRUCT*), we used an additional reference anatomical structure *RefSTRUCT*+, which was significantly closer to the target area. This anatomical landmark varied based on the target position within the heart and also based on regional image quality in all relevant sample CT series. Final products in terms of GDM method, i.e., _1_PTV_GDM_ (*RefSTRUCT*) and _2_PTV_GDM_ (*RefSTRUCT*+) target volumes, show the largest similarity parameter of all mutual comparisons performed as seen in Table 4-line 3 (DICE: mean 0.89, range 0.86–0.94). This is one reason why we believe that using the LMCA is reasonably justified for the estimation of the deformation between the marker and target volume because using *RefSTRUCT*+, which is closer to the target compared with *RefSTRUCT*, did not lead to a significantly different target volume. In addition, a good similarity between DIR-based (PTV_MIM_ and particularly PTV_VELO_) and _1_PTV_GDM_ (DICE range 0.80–0.92, Table 4-line 8) and particularly _1_PTV_GDM−SUM_ (DICE range 0.85–0.91, Table 4-line 9) volumes supports interpretation of the GDM method as a reasonably justified approximation since automated DIR software always considered the entire heart volume as a minimum, i.e., not only the LMCA (*RefSTRUCT*) or an alternative manually selected anatomical surrogate (*RefSTRUCT*+). This means that using the GDM method based on the LMCA surrogate, we obtained similar final outcomes (target volumes) as using a DIR software taking into account at least (deformation of) the entire heart. Nevertheless, before using the GDM method based on the LMCA, we recommend the assessment of its target representativeness on an individual basis and, in case of doubt, consider an alternative anatomical landmark in closer proximity to the target.

Regarding intra- and inter-observer variabilities, the 2-mm resulting isotropic ITV-PTV margin is considered also to cover residual geometry uncertainty aspects. For our technology, it is, namely, LED marker correlation uncertainty. However, since in GDM-based volume, cardiac motion should be included, the same factor causing the increase in the correlation uncertainty (7) should not be included again. Again, for this reason, we decided to use a 2-mm isotropic margin overall. Very similar results between intra- (mean DICE 0.88) and inter-observer variabilities (mean DICE 0.89) demonstrated a good implementation of related work instruction. It is also expected that with time and increasing experience, the variability may decrease with possible margin reduction.

Regarding mutual volume comparisons, using the DICE similarity coefficient is associated with a question of what ranges of values represent “rather similar” and how much “rather different”. The AAPM guidelines (17) contain recommended values that are used to test DIR performance are 0.8–0.9. Also, based on the results of observer reproducibility tests, we consider 0.80 as the minimum for “rather similar” and 0.70 as maximum for “rather different.”

As seen in Table 3, the absolute volumes of the GDM (and DIR) based volumes are larger than the original PTVs built using generic a 3-mm isotropic margin. Direct comparison is not 100% fair owing to individual doctor's intervention at the end of the original target definition process. Where applicable, this may include removing vessels from the target and other volume reduction related to toxicity control. Alternative GDM (and DIR)-based volumes presented in this study did not involve any of such volume reductions so, in many cases, the real difference may be smaller. At the same time, having relatively larger volumes justified by individual motion management leads to higher confidence in the final target volume reductions for mentioned toxicity control reasons. Nevertheless, the relative increase in clinically applied target volumes must be investigated in terms of the potential increase of toxicity and is subjected to ongoing study.

The next relevant aspect is that in this study, we assumed a priory that the general principle of radiation oncology which is that “each part of target must receive the prescription dose” applies to STAR. This normally requires minimum of 95–98% PTV coverage. However, as STAR is not oncology and the biological objective of irradiation is different, this requirement may not be as essential. If it shows that partial coverage of a precisely defined target volume is sufficient, the situation is different. Nevertheless, target volumes considering individual patient deformation due to cardiac and respiratory motion are still more valid compared to volumes based on a generic isotropic margin, especially considering an initial CTV defined at the extreme respiratory phase (*CTebh*).

Overall, the relative increase of absolute target volume does not necessarily mean a final increase in treatment toxicity. The GDM-based volume reflects the center of target location better. This is certainly an advantage even for approaches where partial target coverage would be considered sufficient.

Comparisons between GDM-SUM and DIR-based volumes show similarity supporting expected mutual confirmation of “deformation considered” target volumes. Results from Velocity^®^ show generally larger DICE values compared to results from MIM^®^ however, this should not be interpreted as a result of comparing the two commercial platforms as the purpose and test design were aimed at presenting examples of constructing target volume equivalent in principle with GDM-based volumes using standard clinical software. DIR workflows were neither optimized nor controlled to a degree sufficient for comparing quality of DIR products. User experience and chosen workflow may have an impact on the values obtained.

### Possible Alternatives

Regarding possible alternative approaches, another logical approach when considering deformation for target definition is applying an initial CTV definition process (based on CARTO physiological mapping) to all secondary CT image series, followed again, by merging resulting subvolumes (including the aspect of potential undersampling). However, based on the publications, the method seems not to be sufficiently developed yet for, e.g., relatively large reported inter-observer variability (22) and likely deformation between CARTO surfaces and segmented CT anatomy to match. Repeating the whole target definition process for all motion sampling CT series using the current state of the process would be probably too laborious and affected by observer variations. In addition, IV contrast importance for CARTO transfer would probably need to be revised.

Taking into account the treatment modalities not based on target tracking technology, beam gating technology and even abdominal press limiting respiratory motion range without beam control during treatment also belong to STAR treatment platforms currently in use (4, 5). Comparing relative advantages and disadvantages among treatment platforms is and will be subjected to ongoing studies and further research.

The proposed GDM method is a simple way to account for marker–target deformation-related uncertainty for tracking with Cyberknife^®^ and better control of risk of target underdose. The principle would equally apply to general radiotherapy.

## Data Availability Statement

The raw data supporting the conclusions of this article will be made available by the authors, without undue reservation.

## Ethics Statement

The studies involving human participants were reviewed and approved by IRB University Hospital Ostrava. The patients/participants provided their written informed consent to participate in this study.

## Author Contributions

PD designed the GDM workflow and methods of its validation, participated in data collection including image registrations including MIM software, data transfers, data analysis, and presenting results. LK participated in the study design and methods, data collection including registrations for intra- and inter-observer variabilities, data analysis, and presenting results. DD performed deformable image registrations using velocity. PB participated in data collection, data transfers, and analysis. JC participated in the study design, data collection, and carried out a critical review of the manuscript. All authors commented on previous versions of the manuscript. All authors read and approved the final manuscript.

## Funding

This study was supported by Ministry of Health, Czech Republic—conceptual development of research organization RVO-FNOs/2018, by grant project AZV NU20-000244 from the Ministry of Health of the Czech Republic, and by funding from European Union's Horizon 2020 research and innovation program under grant agreement 945119. The authors have reported that they have no relationships relevant to the contents of this paper to disclose.

## Conflict of Interest

The authors declare that the research was conducted in the absence of any commercial or financial relationships that could be construed as a potential conflict of interest.

## Publisher's Note

All claims expressed in this article are solely those of the authors and do not necessarily represent those of their affiliated organizations, or those of the publisher, the editors and the reviewers. Any product that may be evaluated in this article, or claim that may be made by its manufacturer, is not guaranteed or endorsed by the publisher.

## References

[1] van der Ree MH Blanck O Limpens J Lee CH Balgobind B Dieleman EM . Cardiac radioablation-a systematic review. Heart Rhythm. (2020) 17:1381–92. 10.1016/j.hrthm.2020.03.013 32205299

[2] Lydiard S Blanck O Hugo G O'Brien R Keall P. A review of cardiac radioablation (CR) for arrhythmias: procedures, technology, and future opportunities. Int J Radiat Oncol Biol Phys. (2021) 109:783–800. 10.1016/j.ijrobp.2020.10.036 33160007

[3] Fahimian BP Loo BW Soltys SG Zei P Lo AT Maguire PJ . First in-human stereotactic arrhythmia radioablation (STAR) of ventricular tachycardia: dynamic tracking delivery analysis and implications. Int J Radiat Oncol Biol Phys. (2015) 93:E466–7. 10.1016/j.ijrobp.2015.07.1738

[4] Cvek J Neuwirth R Knybel L Molenda L Otahal B Pindor J . Cardiac radiosurgery for malignant ventricular tachycardia. Cureus. (2014) 6:e190. 10.7759/cureus.190

[5] Wei C Qian PC Boeck M Bredfeldt JS Blankstein R Tedrow UB . Cardiac stereotactic body radiation therapy for ventricular tachycardia: current experience and technical gaps. J Cardiovasc Electrophysiol. (2021) 32:2901–14. 10.1111/jce.15259 34587335

[6] Guckenberger M Baus WW Blanck O Combs SE Debus J Engenhart-Cabillic R et al. Definition and quality requirements for stereotactic radiotherapy: consensus statement from the DEGRO/DGMP working group stereotactic radiotherapy and radiosurgery. Strahlentherapie und Onkologie. (2020) 196:417–20. 10.1007/s00066-020-01603-1 32211940 PMC7182610

[7] Knybel L Cvek J Neuwirth R Jiravsky O Hecko J Penhaker M . Real-time measurement of ICD lead motion during stereotactic body radiotherapy of ventricular tachycardia. Rep Pract Oncol Radiothe. (2021) 26:128–37. 10.5603/RPOR.a2021.0020 34046223 PMC8149135

[8] Neuwirth R Cvek J Knybel L Jiravsky O Molenda L Kodaj M . Stereotactic radiosurgery for ablation of ventricular tachycardia. EP Europace. (2019) 21:1088–95. 10.1093/europace/euz133 31121018

[9] Haskova J Peichl P Pirk J Cvek J Neuwirth R Kautzner J. Stereotactic radiosurgery as a treatment for recurrent ventricular tachycardia associated with cardiac fibroma. HeartRhythm Case Rep. (2019) 5:44–7. 10.1016/j.hrcr.2018.10.007 30693205 PMC6342609

[10] Lloyd MS Wight J Schneider F Hoskins M Attia T Escott C . Clinical experience of stereotactic body radiation for refractory ventricular tachycardia in advanced heart failure patients. Heart Rhythm. (2020) 17:415–22. 10.1016/j.hrthm.2019.09.028 31585181

[11] Gianni C Rivera D Burkhardt JD Pollard B Gardner E Maguire P . Stereotactic arrhythmia radioablation for refractory scar-related ventricular tachycardia. Heart Rhythm. (2020) 17:1241–48. 10.1016/j.hrthm.2020.02.036 32151737

[12] Loo Jr BW Soltys SG Wang L Lo A Fahimian BP Iagaru A . Stereotactic ablative radiotherapy for the treatment of refractory cardiac ventricular arrhythmia. Circ Arrhythm Electrophysiol. (2015) 8:748–50. 10.1161/CIRCEP.115.002765 26082532

[13] Kikinis R Pieper SD Vosburgh K. 3D slicer: a platform for subject-specific image analysis, visualization, and clinical support. Intraoperative Imaging Image-Guided Therapy. (2014) 3:277–89 10.1007/978-1-4614-7657-3_19

[14] Zhu J Chen X Yang B Bi N Zhang T Men K . Evaluation of automatic segmentation model with dosimetric metrics for radiotherapy of esophageal cancer. Front Oncol. (2020) 10:564737. 10.3389/fonc.2020.564737 33117694 PMC7550908

[15] Huttenlocher DP Klanderman GA Rucklidge WJ. Comparing images using the hausdorff distance. IEEE Trans Pattern Anal and Mac Intell. (1993) 15:850–63. 10.1109/34.232073

[16] Lawson JD Schreibmann E Jani AB Fox T. Quantitative evaluation of a cone-beam computed tomography–planning computed tomography deformable image registration method for adaptive radiation therapy. J Appl Clin Med Phys. (2007) 8:96–113. 10.1120/jacmp.v8i4.2432 18449149 PMC5722621

[17] Brock KK Mutic S McNutt TR Li H Kessler ML. Use of image registration and fusion algorithms and techniques in radiotherapy: report of the AAPM radiation therapy committee task group no. 132. Med Phys. (2017) 44:e43–76. 10.1002/mp.12256 28376237

[18] Blanck O Ipsen S Chan MK Bauer R Kerl M Hunold P . Treatment planning considerations for robotic guided cardiac radiosurgery for atrial fibrillation. Cureus. (2016) 8:e705. 10.7759/cureus.705 27588226 PMC4999353

[19] Meroni R. Francesco La Torre (Accuray Inc.): personal communication. (2021).

[20] Speight R Sykes J Lindsay R Franks K Thwaites D. The evaluation of a deformable image registration segmentation technique for semi-automating internal target volume (ITV) production from 4DCT images of lung stereotactic body radiotherapy (SBRT) patients. Radiother Oncol. (2011) 98:277–83. 10.1016/j.radonc.2010.12.007 21257217

[21] Tong Y Yin Y Cheng P Gong G. Impact of deformable image registration on dose accumulation applied electrocardiograph-gated 4DCT in the heart and left ventricular myocardium during esophageal cancer radiotherapy. Radiat Oncol. (2018) 13:1–7. 10.1186/s13014-018-1093-z 30097045 PMC6086020

[22] Abdel-Kafi S Sramko M Omara S de Riva M Cvek J Peichl P . Accuracy of electroanatomical mapping-guided cardiac radiotherapy for ventricular tachycardia: pitfalls and solutions. EP Europace. (2021) 23:1989–97. 10.1093/europace/euab195 34524422

